# The mortality risk factor of community acquired pneumonia patients with chronic obstructive pulmonary disease: a retrospective cohort study

**DOI:** 10.1186/s12890-018-0587-7

**Published:** 2018-01-22

**Authors:** Ruo-Xuan Dai, Qing-Hua Kong, Bei Mao, Wen Xu, Ru-Jia Tao, Xiao-Ru Wang, Qing-Yao Kong, Jin-Fu Xu

**Affiliations:** 1 Department of Respiratory and Critical Care Medicine, Shanghai Pulmonary Hospital, Tongji University School of Medicine, No. 507 Zhengmin Road, Shanghai, 200433 China; 2 Department of Respiratory and Critical Care Medicine, Shanghai Dahua Hospital, No. 901 Old Humin Road, Shanghai, China; 3 Department of Anesthesia and Critical Care, the University of Chicago, Chicago, IL USA

**Keywords:** Severity scoring systems, Mortality, Community acquired pneumonia, Chronic obstructive pulmonary disease

## Abstract

**Background:**

Chronic obstructive pulmonary disease (COPD) is one of the most common comorbidities in community acquired pneumonia (CAP) patients. We aimed to investigate the characteristics and mortality risk factors of COPD patients hospitalized with CAP.

**Methods:**

A retrospective cohort study was conducted at Shanghai Pulmonary Hospital and Shanghai Dahua Hospital. Clinical and demographic data in patients diagnosed with CAP were collected between January 2015 and June 2016. Logistic regression analysis was performed to screen mortality risk factors of COPD patients hospitalized with CAP.

**Results:**

Of the total 520 CAP patients, 230 (44.2%) patients had been diagnosed comorbid with COPD (COPD-CAP). CAP patients comorbid with COPD patients had higher rate of need for ICU admission (18.3% vs 13.1%) and need for NIMV (26.1% vs 1.4%) than without COPD (nCOPD-CAP). The PSI, CURB-65 and APACHE-II scores in COPD-CAP patients were higher than that in nCOPD-CAP patients (95 vs 79, *P* < 0.001; 1 vs 1, *P* < 0.001; 13 vs 8, *P* < 0.001, respectively). Logistic regression analysis indicated that aspiration, D-dimer > 2.0 μg/mL and CURB-65 ≥ 3 were risk factors associated with in-hospital mortality ((odd ratio) OR = 5.678, OR = 4.268, OR = 20.764, respectively) in COPD-CAP patients. The risk factors associated with 60-day mortality in COPD-CAP patients were comorbid with coronary heart disease, aspiration, need for NIMV (non-invasive mechanical ventilation) and CURB-65 ≥ 3 (OR = 5.206, OR = 7.921, OR = 3.974, OR = 18.002, respectively).

**Conclusions:**

COPD patients hospitalized with CAP had higher rate of need for NIMV, need for ICU admission and severity scores than those without COPD. Aspiration, D-dimer > 2.0 μg/mL, comorbid with coronary heart disease, need for NIMV and CURB-65 ≥ 3 were mortality risk factors in CAP patients comorbid with COPD.

**Electronic supplementary material:**

The online version of this article (10.1186/s12890-018-0587-7) contains supplementary material, which is available to authorized users.

## Background

Community acquired pneumonia (CAP) is a common disease associated with high morbidity, mortality and inpatients care costs [1–3]. The 2009–2014 British Thoracic Society (BTS) Audit Programme indicates that the overall 30-day inpatients mortality is 18.0% [4]. Chronic obstructive pulmonary disease (COPD) is a disease with persistent airflow limitation and chronic inflammatory response, which progresses slowly [5]. The number of COPD cases in China increases dramatically from 32.4 million in 1990 to 54.8 million in 2013, which poses a heavy economic burden in China [6, 7].

COPD is one of the most common comorbidities in CAP. In the U.S., from 2005 to 2007, the incidence of inpatients primary pneumonia among elders with COPD is 54.2/1000 person-years, which is nearly seven times higher than that in elders without COPD [8]. Fine M. J. et al. derived a prediction rule to evaluate 30-day mortality in patients with CAP, chronic pulmonary diseases were excluded as a risk factor [9]. Meanwhile, previous study demonstrated that COPD patients hospitalized with CAP had the same mortality rate and demographic features, except for age and gender, as patients without COPD [10]. On the contrary, Restrepo M. I. et al. studied CAP population and indicated that COPD patients hospitalized with CAP had higher 30- and 90-day mortality than patients without COPD [11]. Besides, Guertler C. et al. reported that COPD was one of the risk factors for long-term mortality in CAP patients during 18-month follow-up [12]. Since the influences of COPD on severity scoring and mortality in CAP are controversial, we want to make clear that whether COPD patients hospitalized with CAP have higher severity scores and higher mortality rate. Meanwhile the mortality risk factors for COPD patients hospitalized with CAP still need to be further studied.

The aim of the current study is to investigate the characteristics of CAP patients comorbid with and without COPD, and to find out the mortality risk factors in COPD patients hospitalized with CAP.

## Methods

### Study design and inclusion and exclusion criteria

To perform a retrospective cohort study, we recruited patients with the diagnosis of CAP who were admitted in hospital between January 2015 and June 2016.

Patients were included if they were: 1) age ≥ 18 years; 2) symptoms of an acute lower respiratory tract illness (cough and at least one other lower respiratory tract symptom); 3) new focal chest signs on examination; 4) at least one systemic feature (either a symptom complex of sweating, fevers, shivers, aches and pains and/or temperature of 38°C or more); 5) no other explanation for the illness, which is treated as CAP with antibiotics [3]. Patients were not eligible for inclusion if they had: 1) been diagnosed with asthma, bronchiectasis, interstitial lung disease or other pulmonary infectious diseases; 2) life-threatening diseases (i. e. advanced malignant tumor, severe cardiopulmonary dysfunction, renal or liver dysfunction, etc.) within the past year; 3) severe immunosuppressive such as HIV infection, using longtime high dose of immunosuppressive agents, chemotherapy or organ transplantation; 4) research-related data missing. We only used the first hospitalization information if patients were admitted more than once during our study period.

### Definitions

Diagnosis of COPD was made in patients with clinical symptom (dyspnea, chronic cough or sputum production) and smoking history at least 10 years, and it was confirmed by spirometric evidence of airflow obstruction in stable stage (post-bronchodilator FEV_1_/FVC < 0.70) [5]. The classification of COPD severity was based on the Global Initiative for Chronic Obstructive Lung Disease (GOLD) criteria: GOLD stage I was defined as a forced expiratory volume in 1 s (FEV_1_) greater than 80% predicted, GOLD stage II as FEV_1_ between 50 and 80% pred., GOLD stage III as FEV_1_ between 30 and 50% pred. and GOLD stage IV less than FEV_1_ 30% pred.. Aspiration pneumonia was applied to situations where a patient with risk factors including altered level of consciousness, neurological disorders such as stroke, presence of dysphagia, gastric disorders such as gastro-oesophageal reflux and setting up gastric tube [3]. Need for ICU admission was applied to situations when a patient met the criteria of severe pneumonia and admission to ICU was necessary [1]. Need for non-invasive mechanical ventilation (NIMV) was applied to situations when a patient met one of these criteria, diagnosed COPD with a respiratory acidosis pH 7.25–7.35 (H^+^ 45–56 nmol/l), hypercapnic respiratory failure secondary to chest wall deformity (scoliosis, thoracoplasty) or neuromuscular diseases, cardiogenic pulmonary edema unresponsive to continuous positive airway pressure (CPAP), weaning from tracheal intubation [13]. In-hospital mortality and 60-day mortality were categorized as primary outcomes and need for ICU admission and need for NIMV were categorized as secondary outcomes.

### Severity scoring

Severity of CAP at admission was calculated according to the PSI, CURB-65 and APACHE-II scores. The PSI score is a 20-point scoring system which classifies patients into five risk categories [9]. The CURB-65 score consists of 5 points: confusion, blood urea nitrogen > 7 mmol/L, respiratory rate > 30 breaths/min, systolic blood pressure < 90 mmHg and/or diastolic blood pressure ≤ 60 mmHg, and age ≥ 65 yrs. [14]. The APACHE-II is a scoring system including initial age, previous health status and value of 12 routine physiologic measurements [15]. We categorized the severity of pneumonia into three classes. PSI score: mild, < 90; moderate, 90–130; severe, > 130. CURB-65 score: mild, 0–1; moderate, 2; severe, ≥3. APACHE-II score: mild, 0–9; moderate, 10–19; severe, ≥20.

### Data collection

All data were independently collected by Dr. RXD and Dr. QHK retrospectively according to the inclusion criteria (1) age ≥ 18 years; 2) diagnosed with CAP on admission) with unified Excel table. The unified Excel table was designed by Dr. RXD and Dr. QHK, it included demographic data (gender, age, smoking status), comorbidities (diabetes, coronary heart disease, hypertension, cerebral infarction, asthma, bronchiectasis, interstitial lung diseases, pulmonary infectious diseases, tumor), physical examination (mental status, respiratory rate, pulse, blood pressure and body temperature), laboratory examination, lung function test and X-ray/chest radiographic reports. The severity scores were assessed using PSI, CURB-65 and APACHE-II scores by two researchers. The highest score within 72 h of admission was chosen and recorded. The in-hospital mortalities were assessed by medical records and 60-day mortalities were assessed by telephone survey.

### Statistical analysis

Quantitative data, which were normally distributed, were analyzed with the Student’s t-test. Mann–Whitney U-test was used for calculating differences between data of two groups which were not normally distributed. For qualitative data, the χ^2^ test were used to assess differences. Covariates were recruited in the univariate analysis with in-hospital mortality and 60-day mortality as outcomes. Only when the covariates with a significant (*P* < 0.05) by univariate analysis were entered in the multivariate analysis. Multicollinearity was screened using Spearman rank correlation matrices when variables with |r| > 0.7. A backward stepwise approach in the multivariate analysis was considered to keep significant covariates with a cut-point of 0.05. The discriminatory values of PSI, CURB-65 and APACHE-II scores in relation to in-hospital mortality and 60-day mortality were analyzed by the receiver-operating characteristic (ROC) analysis. ROC curves were performed to obtain the area under the curves (AUCs), cut-off values, sensitivities, specificities. The diagnostic performance was referred as high (AUC > 0.9), moderate (AUC = 0.7–0.9), or low (AUC = 0.5–0.7). The optimal cut-off values were determined by the Youden index (maximum of sensitivity + specificity – 1) (Additional file 1: Figure S1). AUCs were compared among PSI, CURB-65 and APACHE-II using the method of DeLong et al. [16]. All the data were analyzed with SPSS Version 20.0 software (SPSS, Chicago, IL) and Medcalc Version 15.2.2 software (Mariakerke, Belgium).

## Results

### Patient characteristics

**Fig. 1:**
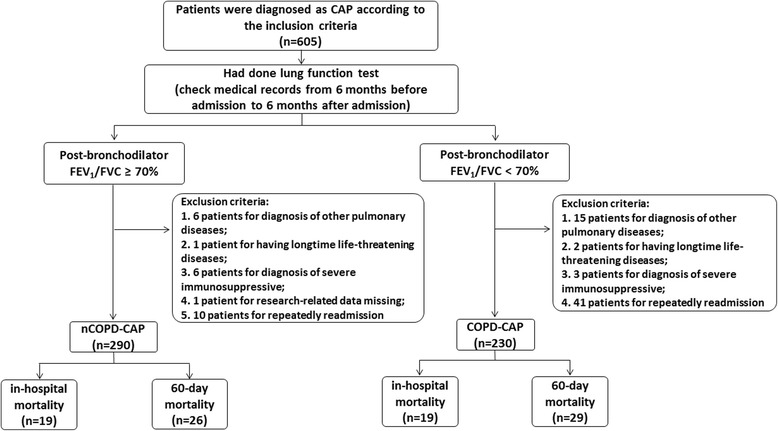
Study flow diagram

Among 605 CAP patients recruited in this study, 291 patients were diagnosed with COPD. 230 (44.2%) patients had been diagnosed comorbid with COPD (COPD-CAP), the recruiting procedure was described in Fig. 1. COPD patients hospitalized with CAP were significantly more likely to be older males and smokers (Table 1). Whereas CAP patients without COPD (nCOPD-CAP) showed higher rates of cough, fever and purulent expectoration. In addition, COPD-CAP patients were significantly more likely to be dyspnea and tachypnea. As for laboratory examinations, COPD-CAP patients presented more acidosis, hypoxemia and hypercapnia, and higher hemoglobin and hematocrit level than nCOPD-CAP. We also did arterial blood gas analysis of COPD-CAP patients during stable stage, and the records were collected in Additional file 2: Table S1. 24 (10.4%) COPD-CAP patients in stable stage had type 2 respiratory failure, less than on admission (52/22.6%). As a retrospective study, patients with post-bronchodilator FEV_1_/FVC ≥ 70% were recruited in nCOPD-CAP group. Therefore, nCOPD-CAP patients in this study had normal lung function. Whereas in COPD-CAP, most patients had severe airflow obstruction (mean pre FEV_1_% = 45.01, SD = 15.76). COPD-CAP patients had higher rate of need for NIMV, need for ICU admission and higher length of hospital stay than those without COPD. Although the mortality in COPD-CAP patients was higher than that in nCOPD-CAP patients, the difference in discriminate value between COPD-CAP and nCOPD-CAP was not significant.

**Table 1:** Baseline characteristics of CAP patients

Covariates	nCOPD-CAP	COPD-CAP	*P* Value^a^
Subjects, n	290	230	
Males	117(40.3)	170(73.9)	**< 0.001**
Age, years	79[67,86]	82[74,87]	**0.003**
Ex-smoker or current smoking	86(29.7)	162(70.4)	**< 0.001**
Comorbidities			
Diabetes	50(17.2)	47(20.4)	0.353
Coronary heart disease	74(25.5)	93(40.4)	**< 0.001**
Hypertension	145(50)	98(42.6)	0.093
Cerebral infarction	50(17.2)	10(4.3)	**< 0.001**
Symptom			
Cough	288(99.3)	209(90.9)	**< 0.001**
Fever ≥38 °C	154(53.1)	79(34.3)	**< 0.001**
Dyspnea	52(17.9)	205(89.1)	**< 0.001**
Expectoration	287(99)	226(98.3)	0.705
Mucoid	66(22.8)	90(39.1)	**< 0.001**
Purulent	211(73.5)	129(57.1)	
Haemoptysis	10(3.4)	7(3)	
Pleural pain	24(8.3)	13(5.7)	0.304
Altered mental state	17(5.9)	9(3.9)	0.418
Respiratory frequency > 30 breaths · min^−1^	11(3.8)	21(9.1)	**0.016**
Laboratory results			
Hemoglobin, g/L	120[108,130.25]	124[113.75,135]	**0.001**
Hematocrit, %	36.15[32.78,39]	37.7[34.5,41]	**< 0.001**
WBC count > 10 × 10^9^ cell	84(29.0)	79(34.3)	0.189
C-reactive protein, mg/L	24.25[5.08,70.57]	32.67[7.99,72.55]	0.267
Arterial PH < 7.35	12(4.1)	58(25.2)	**< 0.001**
PaO_2_ < 60 mmHg	44(15.2)	81(35.2)	**< 0.001**
PaCO_2_ > 50 mmHg	16(5.5)	74(32.2)	**< 0.001**
Albumin, g/L	34.35[31.2,37.35]	34.1[31.28,37.3]	0.728
Pre-albumin, mg/L	146.779 ± 65.414	145.974 ± 65.850	0.889
D-dimer > 2.0 μg/mL	62(21.4)	45(19.6)	0.611
Spirometry			
FEV_1_% predicted	≥70	45.01 ± 15.76	
GOLD 1^b^		9(3.9)	
GOLD 2		80(34.8)	
GOLD 3		88(38.3)	
GOLD 4		53(23)	
Aspiration	53(18.3)	29(12.6)	0.078
Need for ICU admission	38(13.1)	42(18.3)	0.105
Need for NIMV	4(1.4)	60(26.1)	**< 0.001**
Outcome measures			
Length of hospital stay, d	14[12,19]	16[13.88,20.63]	**< 0.001**
In-hospital mortality	19(6.6)	19(8.3)	0.457
60-day mortality	26(9)	29(12.6)	0.180

Data given as n(%) or mean ± SD or median [interquartile range]

*CAP* community acquired pneumonia, *COPD* chronic obstructive pulmonary disease, *nCOPD-CAP* CAP patients without COPD, *COPD-CAP* CAP patients with COPD, *ICU* intensive care unit, *NIMV* non-invasive mechanical ventilation, *WBC* white blood cell, *PaO*_*2*_ partial pressure of arterial oxygen, *PaCO*_*2*_ partial pressure of arterial carbon dioxide, *FEV*_*1*_ forced expiratory volume in one second

^a^Values in bold indicate *P* < 0.05

^b^GOLD I was defined as FEV_1_/FVC ratio < 70% and FEV_1_ > 80% predicted; GOLD II as FEV_1_/FVC ratio < 70% and FEV_1_ 50%–80% predicted; GOLD III as FEV_1_/FVC ratio < 70% and FEV_1_ 30%–50% predicted; GOLD IV as FEV_1_/FVC ratio < 70% and FEV_1_ < 30% predicted

### Comparison of different primary outcomes of predictive rules

The COPD-CAP patients were more severe than nCOPD-CAP patients in PSI, CURB-65 and APACHE-II severity score systems (79 [64.75, 101.25] vs 95 [77,128.25], *P* < 0.001; 1 [1, 2] vs 1 [1, 2], *P* < 0.001; 8 [6, 12] vs 13 [9.75,17], *P* < 0.001) (Table 2). As for in-hospital mortality and 60-day mortality, only the constituent ratio (mild, moderate, severe) of APACHE-II score was statistically significant.

**Table 2:** Comparison of primary outcomes of predictive rules

Risk groups	No of patients (*N* = 520)			In-hospital mortality (*N* = 38)			60-day mortality (*N* = 55)		
	nCOPD-CAP	COPD-CAP	P value^a^	nCOPD-CAP	COPD-CAP	P value^a^	nCOPD-CAP	COPD-CAP	**P value** ^**a**^
PSI	79[64.75,101.25]	95[77.128.25]	**< 0.001**	169.26 ± 31.52	170.37 ± 46.21	0.932	160.42 ± 32.1	164.93 ± 41.11	0.655
Mild	184(63.4)	106(46.1)	**< 0.001**	0(0)	1(5.3)	0.171	0(0)	1(3.4)	0.907
Moderate	77(26.6)	68(29.6)		2(10.5)	4(21)		5(19.2)	6(20.7)	
Severe	29(10)	56(24.3)		17(89.5)	14(73.7)		21(90.8)	22(75.9)	
CURB-65	1[1,2]	1[1,2]	**< 0.001**	3.47 ± 0.84	3.37 ± 1.21	0.758	3.12 ± 0.99	3.10 ± 1.08	0.966
Mild	214(73.8)	120(52.2)	**< 0.001**	0(0)	2(10.5)	0.2	1(3.8)	2(6.9)	0.929
Moderate	53(18.3)	78(33.9)		1(5.3)	2(10.5)		5(19.2)	6(20.7)	
Severe	23(7.9)	32(13.9)		18(94.7)	15(79)		20(76.9)	21(72.4)	
APACHE-II	8[6,12]	13[9.75,17]	**< 0.001**	28[26,32]	25[15,33]	0.253	26.62 ± 5.14	24.38 ± 8.12	0.234
Mild	182(62.8)	57(24.8)	**< 0.001**	0(0)	0(0)	**< 0.001**	0(0)	0(0)	**< 0.001**
Moderate	77(26.5)	134(58.2)		0(0)	6(31.6)		1(3.8)	9(31)	
Severe	31(10.7)	39(17)		19(100)	13(68.4)		25(96.2)	20(69)	

Data given as n(%) or mean ± SD or median [interquartile range]

*P* value^a^ (Student’s t test and Mann–Whitney U test for PSI, CURB-65 or APACHE-II; χ^2^ test for mild, moderate and severe of PSI, CURB-65 or APACHE-II; Values in bold indicate *P* < 0.05)

### Mortality risk factors

Thirteen covariates were recruited in the univariate analysis with in-hospital mortality and 60-day mortality as outcomes. Only 3 covariates had statistically significant difference (*P* < 0.05) in logistic regression of in-hospital mortality (Table 3). Aspiration (OR = 5.678, 95%CI = 1.590–20.277, *P* = 0.008), D-dimer > 2.0 μg/mL (OR = 4.268, 95%CI = 1.205–15.124, *P* = 0.025) and CURB-65 ≥ 3 (OR = 20.764, 95%CI = 5.632–76.559, *P* = < 0.001) were prognostic predictors of in-hospital mortality in CAP patients with COPD. Meanwhile, coronary heart disease (OR = 5.206, 95%CI = 1.531–17.703, *P* = 0.008), aspiration (OR = 7.921, 95%CI = 2.256–27.816, *P* = 0.011), need for NIMV (OR = 3.974, 95%CI = 1.245–12.688, *P* = 0.020) and CURB-65 ≥ 3 (OR = 18.002, 95%CI = 5.867–55.237, *P* < 0.001) were associated with 60-day mortality (Table 4). Additionally, we screened the risk factors for in-hospital mortality and 60-day mortality in nCOPD-CAP patients using logistic analysis (Additional file 3: Table S2 and Additional file 4: Table S3). Age ≥ 70, coronary heart disease, cerebral infarction, need for NIMV, albumin < 30 g/dl, D-dimer > 2.0 μg/mL, PSI > 130, CURB-65 ≥ 3, APACHE-II ≥ 20 were recruited in multivariate analysis. PSI > 130 (OR = 31.095, 95%CI = 1.443–670.154, *P* = 0.028), CURB-65 ≥ 3 (OR = 51.936, 95%CI = 3.130–861.725, *P* = 0.006) and APACHE-II ≥ 20 (OR = 43.210, 95%CI = 2.705–690.249, *P* = 0.008) were the risk factors for in-hospital mortality. Cerebral infarction (OR = 20.659, 95%CI = 2.001–213.26, *P* = 0.011), PSI > 130 (OR = 12.186, 95%CI = 1.744–85.153, *P* = 0.012) and CURB-65 ≥ 3 (OR =47.999, 95%CI = 2.619–879.551, *P* < 0.001) were the risk factors for 60-day mortality in nCOPD-CAP patients.

**Table 3:** Logistic regression analyses of the risk factors associated with in-hospital mortality in CAP patients with COPD

	Univariate analysis		Multivariate analysis	
	OR (95%CI)	*P* value^a^	OR (95%CI)	*P* value^a^
Age ≥ 70	4.211(0.546,32.475)	0.168		
Ex-smoker or current smoking	0.546(0.209,1.425)	0.216		
Coronary heart disease	2.751(1.040,7.276)	**0.041**		
Cerebral infarction	2.985(0.587,15.185)	0.188		
Aspiration	19.563(6.806,56.228)	**< 0.001**	5.203(1.443,18.757)	**0.012**
Need for NIMV	1.140(0.359,3.620)	0.824		
Albumin< 30 g/dl	4.855(1.833,12.859)	**0.001**		
D-dimer> 2.0 μg/mL	9.247(3.390,25.222)	**< 0.001**	5.026(1.395,18.108)	**0.014**
Arterial PH < 7.35	3.634(1.396,9.461)	**0.008**		
PaCO_2_ > 50 mmHg	2.552(0.990,6.580)	0.053		
PSI > 130	11.267(3.843,33.030)	**< 0.001**		
CURB-65 ≥ 3	42.794(12.771,143.399)	**< 0.001**	23.299(6.246,86.903)	**< 0.001**
APACHE-II ≥ 20	15.417(5.390,44.092)	**< 0.001**		

*OR* odds ratio, *CI* confidence interval, *NIMV* non-invasive mechanical ventilation

^a^Values in bold indicate *P* < 0.05

**Table 4:** Logistic regression analyses of the risk factors associated with 60-day mortality in CAP patients with COPD

	Univariate analysis		Multivariate analysis	
	OR (95%CI)	*P* value^a^	OR (95%CI)	*P* value^a^
Age ≥ 70	6.957(0.919,52.676)	0.060		
Ex-smoker or current smoking	0.648(0.288,1.457)	0.294		
Coronary heart disease	3.896(1.686,9.004)	**< 0.001**	5.206(1.531,17.703)	**0.008**
Cerebral infarction	1.787(0.360,8.860)	0.477		
Aspiration	14.311(5.769,35.501)	**< 0.001**	7.921(2.256,27.816)	**0.001**
Need for NIMV	6.195(2.719,14.116)	**< 0.001**	3.974(1.245,12.688)	**0.020**
Albumin< 30 g/dl	4.024(1.746,9.270)	**< 0.001**		
D-dimer> 2.0 μg/mL	5.118(2.248,11.654)	**< 0.001**		
Arterial PH < 7.35	5.325(2.355,12.025)	**< 0.001**		
PaCO_2_ > 50 mmHg	4.237(1.883,9.534)	**< 0.001**		
PSI > 130	15.437(6.109,39.009)	**< 0.001**		
CURB-65 ≥ 3	45.341(16.413,125.255)	**< 0.001**	18.002(5.867,55.237)	**< 0.001**
APACHE-II ≥ 20	21.287(8.503,53.292)	**< 0.001**		

*OR* odds ratio, *CI* confidence interval, *NIMV* non-invasive mechanical ventilation

^a^Values in bold indicate *P* < 0.05

### ROC curves

The discriminatory ability of PSI, CURB-65 and APACHE-II scores to predict in-hospital mortality and 60-day mortality of COPD-CAP patients were analyzed and compared using areas under receiver operating characteristic (ROCs) curves (Additional file 5: Figure S2). The PSI score had good discriminative ability for death than CURB-65 and APACHE-II scores (AUC_in-hospital mortality_ = 0.896, Se_in-hospital mortality_ = 73.7%, Sp_in-hospital mortality_ = 91.9%; AUC_60-day mortality_ = 0.911, Se_60-day mortality_ = 89.7%, Sp_60-day mortality_ = 77.1%). Whereas, there was no significant difference among PSI, CURB-65 and APACHE-II scores in predicting in-hospital mortality (PSI vs CURB-65, Z statistic = 0.204, *P* = 0.839; PSI vs APACHE-II, Z statistic = 1.542, *P* = 0.123; CURB-65 vs APACHE-II, Z statistic = 0.536, *P* = 0.5920). The differences among PSI, CURB-65 and APACHE-II scores in predicting 60-day mortality were not significant (PSI vs CURB-65, Z statistic = 0.608, *P* = 0.5433; PSI vs APACHE-II, Z statistic = 0.609, *P* = 0.5428; CURB-65 vs APACHE-II, Z statistic = 0.296, *P* = 0.7673). Additionally, we compared secondary outcomes (need for ICU admission and need for NIMV) of predictive rules in Additional file 6: Figure S3. In predicting need for ICU admission, there was a trend toward significance between PSI and APACHE-II (Z statistic = 1.840, *P* = 0.0658), and there was no significance between PSI and CURB-65 (Z statistic = 1.355, *P* = 0.175), and between CURB-65 and APACHE-II (Z statistic = 0.0622, *P* = 0.9504). Moreover, there was a significance between PSI and CURB-65 (Z statistic = 3.411, *P* = 0.0006), and between CURB-65 and APACHE-II (Z statistic = 4.110, *P* < 0.0001) in predicting need for NIMV. There was no significance between PSI and APACHE-II (Z statistic = 0.679, *P* = 0.4969).

## Discussion

In the present study, we showed that COPD patients hospitalized with CAP had higher rate of need for NIMV, need for ICU admission and severity scores than those without COPD. Aspiration, D-dimer > 2.0 μg/mL, comorbid with coronary heart disease, need for NIMV and CURB-65 ≥ 3 were mortality risk factors in CAP patients comorbid with COPD.

We found that COPD was a frequent comorbidity in CAP patients, less than half of the whole population (44.2%). These results were consistence with other researches in which the rates were between 16 and 45.4% [10, 11, 17–20]. In the present study, COPD patients hospitalized with CAP were male, older age and smokers in predominance, and significantly more likely to be dyspnea and tachypnea. In addition, COPD-CAP patients presented more acidosis, hypoxemia and hypercapnia than nCOPD-CAP patients. COPD-CAP patients showed high rate of need for NIMV and need for ICU admission, meanwhile with higher PSI, CURB-65 and APACHE-II scores than nCOPD-CAP patients. We deemed that COPD-CAP patients presented more severe than nCOPD-CAP patients on admission. Therefore, chronic obstructive pulmonary disease should be taken into account as one of the comorbidities in assessment of severity of community acquired pneumonia.

The in-hospital mortality and 60-day mortality of COPD-CAP patients in our study was 8.3 and 12.6%. In a cohort of 262 sample size, the 30-day mortality of CAP patients with COPD was 8.4%, and there was no difference between CAP patient with and without COPD [10]. The same result was found in a cohort with 367 CAP patients [21]. Nevertheless, in the study of Restrepo M. I., COPD patients hospitalized with CAP, compared to patients without COPD, showed significantly higher 30- and 90-day mortality [11]. Three main reasons might account for the same mortality rate between COPD-CAP and nCOPD-CAP patients in our study. First, the CAP patients recruited in this study, no matter nCOPD-CAP or COPD-CAP, they were more likely to have higher mean age, more coexisting illnesses, different distribution of etiology and atypical clinical presentations. Meanwhile, the all-cause mortality, which was used in this study, was significantly related to individual conditions. Secondly, using systemic corticosteroid was found to be associated with decreased mortality of CAP-COPD patients admitted to ICU [22], which might account for same mortality between nCOPD-CAP and COPD-CAP patients. Thirdly, since CAP population was relatively small in this research and previous researches [10, 21], enlarge study population would be available to find the difference of mortality between nCOPD-CAP and COPD-CAP patients.

Few studies paid attention to the mortality risk factors of COPD patients hospitalized with COPD. We found that aspiration was associated with in-hospital mortality and 60-day mortality in COPD-CAP patients. Abnormal swallowing reflexes frequently occurred in COPD patients. Clayton N. A. et al. found that in COPD patients, reduced laryngopharyngeal sensitivity and impaired swallowing function might cause pharyngeal residue and subsequent inhalation of pharyngeal contents [23], which might cause bacterial colonization and recurrence of aspiration [24]. Patients at risk of aspiration pneumonia had poor short-term prognosis and the risk of recurrence of pneumonia [25–27], which in accordance with COPD-CAP population in present study. D-dimer > 2.0 μg/mL were associated with in-hospital mortality in COPD-CAP patients. Coagulation abnormalities were common in CAP patients without organ dysfunction [28]. The endotoxins of pathogens might cause pro-inflammatory states and result in activation of the coagulation cascade and inhibition of fibrinolysis [29, 30], which impacted on the formation of D-dimer. Previous reports found that the level of D-dimer had predictive value for mechanical ventilation therapy and mortality risk (AUC = 0.75, AUC = 0.859) [31, 32]. But D-dimer > 2.0 μg/mL could not be a risk factor for long-term mortality. Incident cardiac complications were associated with increased risk of death at 30 days in CAP patients [33]. In the present study, comorbid with coronary heart disease was one of the risk factors of 60-day mortality in COPD-CAP patients. Previous study indicated that CAP promoted platelet activation and thrombosis, at the same time, created an imbalance between myocardial oxygen supply and demand, which might increase the cardiovascular events [34]. Whereas, in our present study, need for NIMV was associated with 60-day mortality in COPD-CAP patients. Non-invasive positive pressure ventilation was used when CAP patients in acute hypoxemic respiratory failure, which was well tolerated and was associated with a reduction of intubation rate and of stay in ICU [35]. The ICU mortality was 50% for COPD patients with CAP failed non-invasive ventilation [36]. CURB-65 ≥ 3 and PSI > 90 were found to be independent risk factors for case-fatality rate in CAP patients with COPD [20, 37]. In our analysis, CURB-65 ≥ 3, other than PSI > 130 or APACHE-II ≥ 20, was identified to be a high risk factor in relation to in-hospital mortality and 60-day mortality in COPD-CAP patients.

In ROC analysis, all scoring systems in COPD-CAP inpatients had good predictive values in mortality, whereas the predictive abilities among three severity scoring systems had no discrimination. In previous study, it was confirmed that PSI score was the best indicator in elder patients with CAP [38]. Richards G. et al. reported that PSI, CURB-65 and APACHE-II performed similarly in predicting in-hospital mortality and 28-day mortality in CAP population [39]. According to the results of ROC analysis, we thought PSI, CURB-65 and APACHE-II scores performed similarly in predicting in-hospital mortality and 60-day mortality in COPD-CAP inpatients.

There were some limitations that should be acknowledged in this study. Firstly, as a retrospective observational study, we still had a potential selection bias. To tackle these problems, we chose two researchers to collect and organize data independently; patients with indefinite diagnosis were not recruited in this study. Second, this study was performed in only two centers and the sample size was relatively small. Therefore, conducting multicenter study and enlarging sample size would be more available for further research.

## Conclusions

In conclusion, our study demonstrated that COPD patients hospitalized with CAP were older, easier to present respiratory failure. COPD-CAP patients had higher rate of need for NIMV, need for ICU admission and severity scores than those without COPD. Nevertheless, CAP patients comorbid with COPD had the same mortality rate as patients without COPD. Aspiration, D-dimer > 2.0 μg/mL, comorbid with coronary heart disease, need for NIMV and CURB-65 ≥ 3 were mortality risk factors in CAP patients comorbid with COPD.

## Additional files

Additional file 1: Figure S1. Computing processes of Youden index. The optimal Sensitivity and Specificity of each ROC curve are highlighted. (TIFF 3568 kb)

Additional file 2: Table S1. Arterial blood gas analysis of COPD-CAP patients in stable stage and on admission. (DOC 31 kb)

Additional file 3: Table S2. Logistic regression analyses of the risk factors associated with in-hospital mortality in CAP patients without COPD. (DOC 37 kb)

Additional file 4: Table S3. Logistic regression analyses of the risk factors associated with 60-day mortality in CAP patients without COPD. (DOC 38 kb)

Additional file 5: Figure S2. ROC curves for the PSI, CURB-65 and APACHE-II to predict primary outcomes in CAP patients with COPD. (A) ROC curves for the PSI, CURB-65 and APACHE-II to predict in-hospital mortality in CAP patients with COPD. (B) ROC curves for the PSI, CURB-65 and APACHE-II to predict 60-day mortality in CAP patients with COPD. ROC curve, receiver operating characteristic curve; AUC, area under the curve; Se, Sensitivity; Sp, Specificity; CI, confidence interval. (TIFF 881 kb)

Additional file 6: Figure S3. ROC curves for the PSI, CURB-65 and APACHE-II to predict secondary outcomes in CAP patients with COPD. (A) ROC curves for the PSI, CURB-65 and APACHE-II to predict need for ICU admission in CAP patients with COPD. (B) ROC curves for the PSI, CURB-65 and APACHE-II to predict need for NIMV in CAP patients with COPD. ROC curve, receiver operating characteristic curve; ICU, intensive care unit; NIMV, non-invasive mechanical ventilation AUC, area under the curve; Se, Sensitivity; Sp, Specificity; CI, confidence interval. (TIFF 901 kb)

## Abbreviations

APACHE-II: Acute Physiology and Chronic Health Evaluation II
AUC: Area under the curve
BTS: British Thoracic Society
CAP: Community acquired pneumonia
CI: Confidence interval
COPD: Chronic obstructive pulmonary disease
COPD-CAP: CAP patients comorbid with COPD
CPAP: Continuous positive airway pressure
CRP: C-reactive protein
ICU: Intensive care unit
nCOPD-CAP: CAP patients comorbid without COPD
NIMV: Non-invasive mechanical ventilation
OR: Odds ratio
PaCO_2_: Partial pressure of arterial carbon dioxide
PaO_2_: Partial pressure of arterial oxygen
PSI: Pneumonia severity index
ROC curve: Receiver operating characteristic curve
Se: Sensitivity
Sp: Specificity
WBC: White blood cell
