# Supplementary figures and images for: The mortality risk factor of community acquired pneumonia patients with chronic obstructive pulmonary disease: a retrospective cohort study
Source: BMC Pulm Med. 2018 Jan 22;18:12. doi: 10.1186/s12890-018-0587-7 (PMC5778745; doi:10.1186/s12890-018-0587-7)

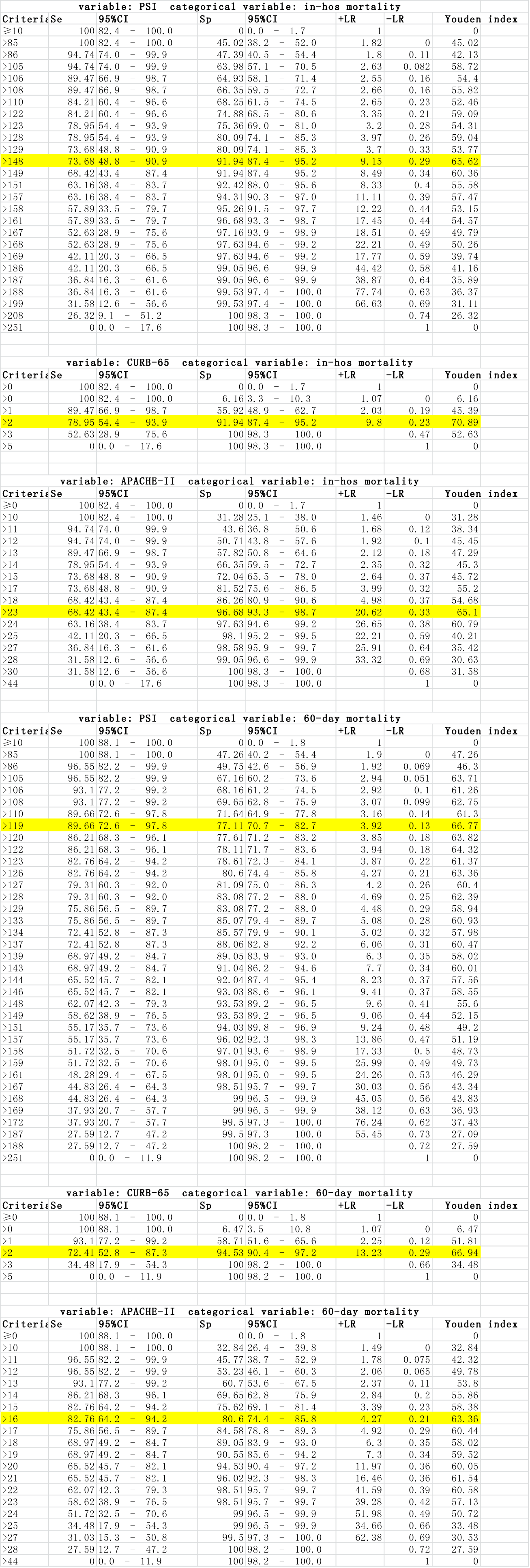

Supplement: Supplementary file 1 — Computing processes of Youden index. The optimal Sensitivity and Specificity of each ROC curve are highlighted. (TIFF 3568 kb) [file 12890_2018_587_MOESM1_ESM.tif]

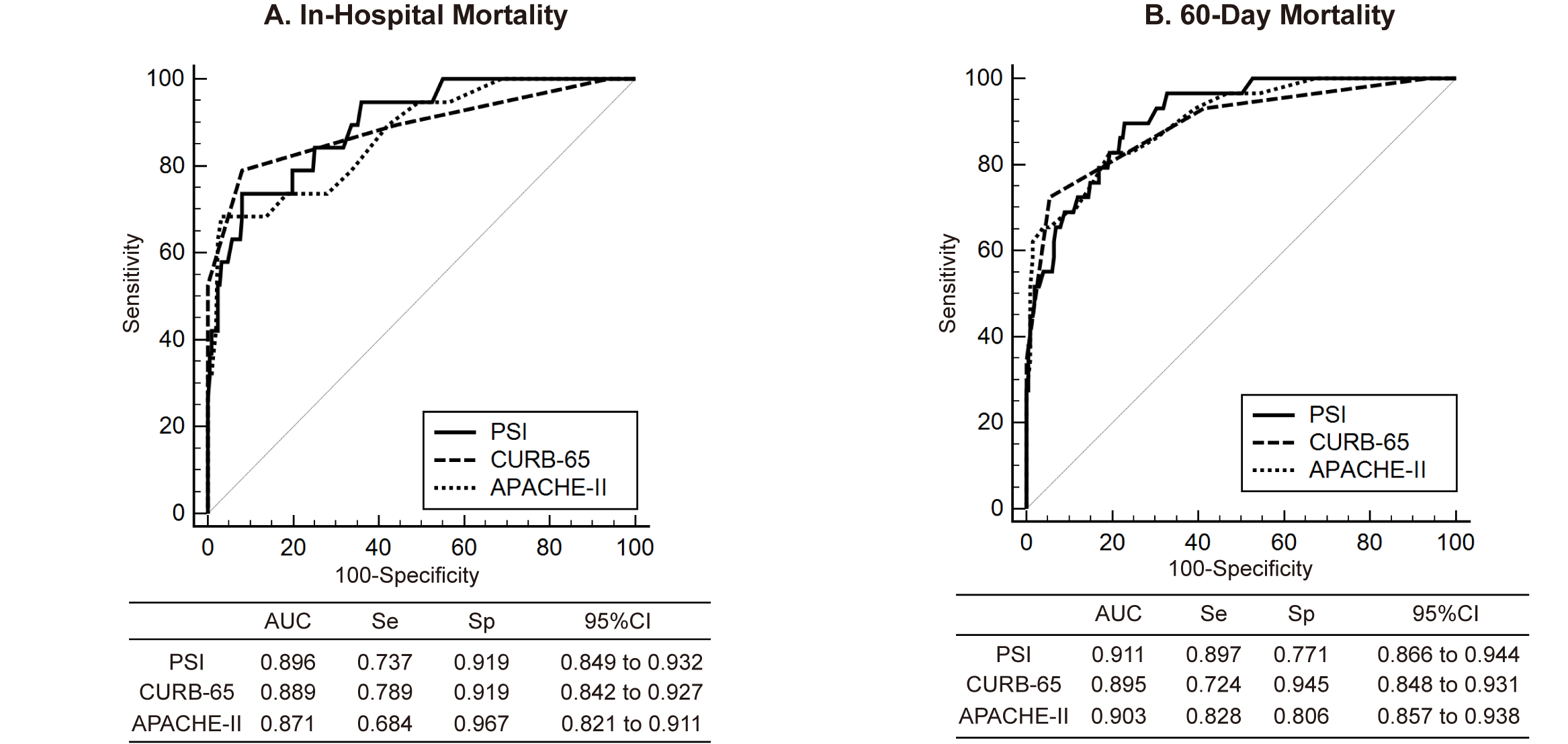

Supplement: Supplementary file 5 — ROC curves for the PSI, CURB-65 and APACHE-II to predict primary outcomes in CAP patients with COPD. (A) ROC curves for the PSI, CURB-65 and APACHE-II to predict in-hospital mortality in CAP patients with COPD. (B) ROC curves for the PSI, CURB-65 and APACHE-II to predict 60-day mortality in CAP patients with COPD. ROC curve, receiver operating characteristic curve; AUC, area under the curve; Se, Sensitivity; Sp, Specificity; CI, confidence interval. (TIFF 881 kb) [file 12890_2018_587_MOESM5_ESM.tif]

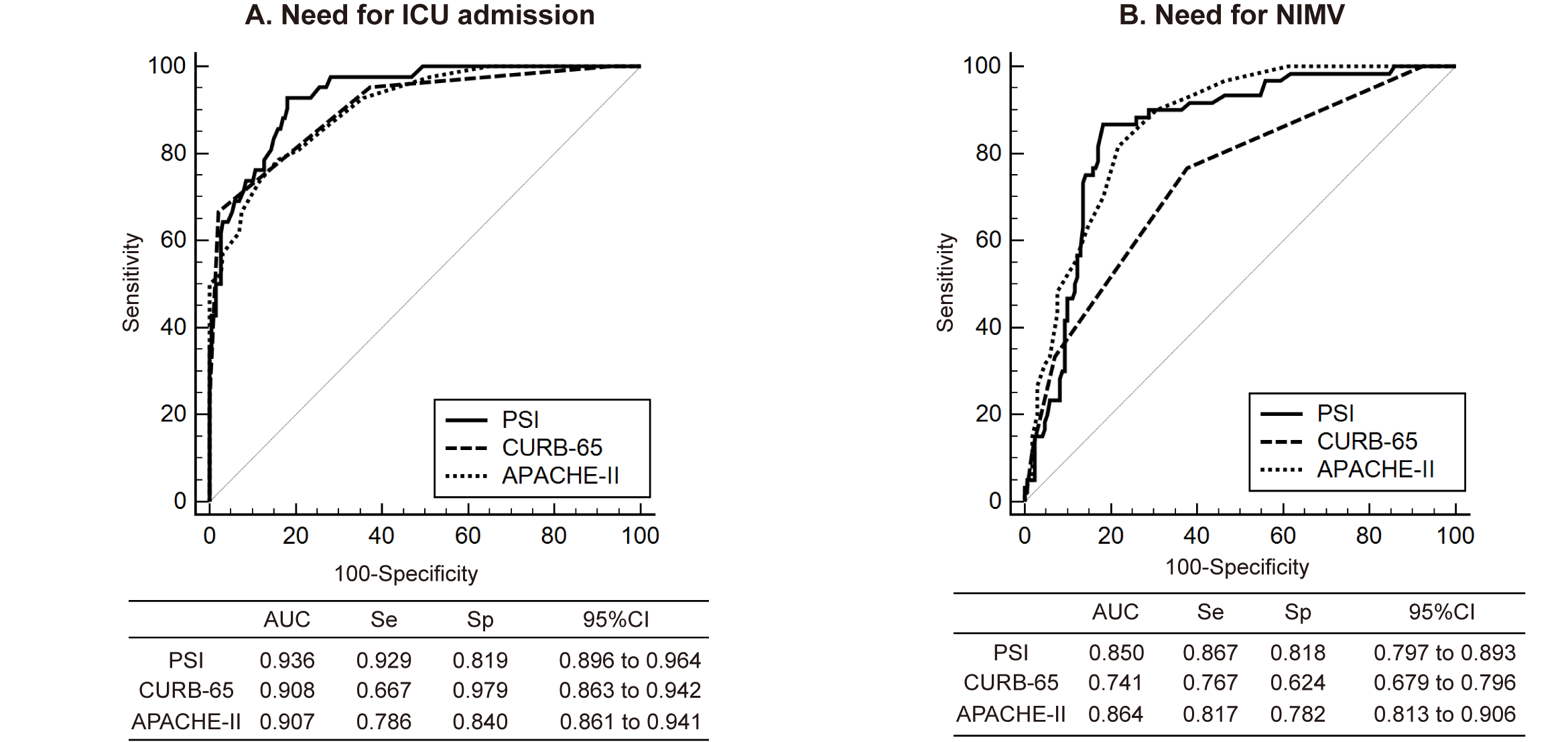

Supplement: Supplementary file 6 — ROC curves for the PSI, CURB-65 and APACHE-II to predict secondary outcomes in CAP patients with COPD. (A) ROC curves for the PSI, CURB-65 and APACHE-II to predict need for ICU admission in CAP patients with COPD. (B) ROC curves for the PSI, CURB-65 and APACHE-II to predict need for NIMV in CAP patients with COPD. ROC curve, receiver operating characteristic curve; ICU, intensive care unit; NIMV, non-invasive mechanical ventilation AUC, area under the curve; Se, Sensitivity; Sp, Specificity; CI, confidence interval. (TIFF 901 kb) [file 12890_2018_587_MOESM6_ESM.tif]
