# Supplementary material for: The mortality risk factor of community acquired pneumonia patients with chronic obstructive pulmonary disease: a retrospective cohort study
Source: BMC Pulm Med. 2018 Jan 22;18:12. doi: 10.1186/s12890-018-0587-7 (PMC5778745; doi:10.1186/s12890-018-0587-7)
Supplement: Supplementary file 2 — Arterial blood gas analysis of COPD-CAP patients in stable stage and on admission. (DOC 31 kb) [file 12890_2018_587_MOESM2_ESM.doc]

**Table S1. Arterial blood gas analysis of COPD-CAP patients in stable stage and on admission**

|  | **Stable stage** | **On admission** | **P value^a^** |
| --- | --- | --- | --- |
| Arterial PH < 7.35 | 17(7.4) | 58(25.2) | **<0.001** |
| PaO_2_ < 60 mmHg | 44(19.1) | 81(35.2) | **<0.001** |
| PaCO_2_ > 50 mmHg | 54(23.5) | 74(32.2) | **0.048** |

Data present as n(%)

^a^Values in bold indicate P < 0.05.
