# Supplementary material for: The mortality risk factor of community acquired pneumonia patients with chronic obstructive pulmonary disease: a retrospective cohort study
Source: BMC Pulm Med. 2018 Jan 22;18:12. doi: 10.1186/s12890-018-0587-7 (PMC5778745; doi:10.1186/s12890-018-0587-7)
Supplement: Supplementary file 3 — Logistic regression analyses of the risk factors associated with in-hospital mortality in CAP patients without COPD. (DOC 37 kb) [file 12890_2018_587_MOESM3_ESM.doc]

**Table S2. Logistic regression analyses of the risk factors associated with in-hospital mortality in CAP patients without COPD**

|  | **Univariate analysis** | | **Multivariate analysis** | |
| --- | --- | --- | --- | --- |
|  | **OR (95%CI)** | **P value^a^** | **OR (95%CI)** | **P value^a^** |
| Age≥70 | 8.802(1.157,66.996) | **0.036** |  |  |
| Ex-smoker or current smoking | 0.838(0.292,2.403) | 0.742 |  |  |
| Coronary heart disease | 2.852(1.111,7.322) | **0.029** |  |  |
| Cerebral infarction | 8.179(3.095,21.617) | **<0.001** |  |  |
| Aspiration | 121.371(15.707,937.842) | **<0.001** |  |  |
| Need for NIMV | 15.824(2.098,119.321) | **0.007** |  |  |
| Albumin<30g/dl | 30.800(8.581,110.553) | **<0.001** |  |  |
| D-dimer>2.0μg/mL | 17.872(5.677,56.269) | **<0.001** |  |  |
| Arterial PH<7.35 | 20.385(5.776,71.946) | **<0.001** |  |  |
| PaCO_2_>50mmHg | 12.046(3.794,38.244) | **<0.001** |  |  |
| PSI>130 | 425.455(51.990,3481.677) | **<0.001** | 31.095(1.443,670.154) | **0.028** |
| CURB-65≥3 | 957.600(106.163,8637.652) | **<0.001** | 51.936(3.130,861.725) | **0.006** |
| APACHE-Ⅱ≥20 | 504.375(85.463,2976.664) | **<0.001** | 43.210(2.705,690.249) | **0.008** |

OR, odds ratio; CI, confidence interval; NIMV, non-invasive mechanical ventilation.

^a^Values in bold indicate P < 0.05.
