# Supplementary material for: The mortality risk factor of community acquired pneumonia patients with chronic obstructive pulmonary disease: a retrospective cohort study
Source: BMC Pulm Med. 2018 Jan 22;18:12. doi: 10.1186/s12890-018-0587-7 (PMC5778745; doi:10.1186/s12890-018-0587-7)
Supplement: Supplementary file 4 — Logistic regression analyses of the risk factors associated with 60-day mortality in CAP patients without COPD. (DOC 38 kb) [file 12890_2018_587_MOESM4_ESM.doc]

**Table S3. Logistic regression analyses of the risk factors associated with 60-day mortality in CAP patients without COPD**

|  | **Univariate** **analysis** | | **Multivariate analysis** | |
| --- | --- | --- | --- | --- |
|  | **OR (95%CI)** | **P value^a^** | **OR (95%CI)** | **P value^a^** |
| Age≥70 | 12.714(1.695,95.367) | **0.013** |  |  |
| Ex-smoker or current smoking | 1.060(0.442,2.539) | 0.896 |  |  |
| Coronary heart disease | 2.340(1.022,5.354) | **0.044** |  |  |
| Cerebral infarction | 10.824(4.542,25.791) | **<0.001** | 20.659(2.001,213.26) | **0.011** |
| Aspiration | 97.241(21.846,432.845) | **<0.001** |  |  |
| Need for NIMV | 34.304(3.429,343.168) | **0.003** |  |  |
| Albumin<30g/dl | 21.111(7.942,56.120) | **<0.001** |  |  |
| D-dimer>2.0μg/mL | 11.250(4.604,27.492) | **<0.001** |  |  |
| Arterial PH<7.35 | 46.059(11.407,185.978) | **<0.001** |  |  |
| PaCO_2_>50mmHg | 10.439(3.502,31.115) | **<0.001** |  |  |
| PSI>130 | 201.929(54.848,743.417) | **<0.001** | 12.186(1.744,85.153) | **0.012** |
| CURB-65≥3 | 290(67.443,1246.984) | **<0.001** | 47.999(2.619,879.551) | **<0.001** |
| APACHE-Ⅱ≥20 | 420.8(50.678,3494.096) | **<0.001** |  |  |

OR, odds ratio; CI, confidence interval; NIMV, non-invasive mechanical ventilation.

^a^Values in bold indicate P < 0.05.
